# Examining the potential contribution of social theory to developing and supporting Australian Indigenous-mainstream health service partnerships

**DOI:** 10.1186/s12939-014-0075-5

**Published:** 2014-09-20

**Authors:** Emma Haynes, Kate P Taylor, Angela Durey, Dawn Bessarab, Sandra C Thompson

**Affiliations:** Western Australian Centre for Rural Health, University of Western Australia, Perth, Australia; Curtin Health Innovation Research Institute, Curtin University, Perth, Australia; Centre for Aboriginal Medical and Dental Health, University of Western Australia, Perth, Australia

**Keywords:** Partnerships, Indigenous health, Social theory, Collaboration, Intercultural

## Abstract

**Introduction:**

The substantial gap in life expectancy between Indigenous and non-Indigenous Australians has been slow to improve, despite increased dedicated funding. Partnerships between Australian Indigenous and mainstream Western biomedical organisations are recognised as crucial to improved Indigenous health outcomes. However, these partnerships often experience challenges, particularly in the context of Australia’s race and political relations.

**Methods:**

We examined the relevant literature in order to identify the potential role for social theory and theoretical models in developing and maintaining intercultural partnerships. Having identified relevant theoretical models, terms and possible key words, a range of databases were searched and relevant articles selected for inclusion. An integrative approach brought together theoretical models and practical considerations about working in partnership, to inform our analysis of the literature.

**Findings:**

Considering partnerships between Australian Indigenous and mainstream health organisations as ‘bi-cultural’ is simplistic: rather they are culturally diverse across social and professional levels. As such, partnerships between Australian Indigenous and mainstream health organisations may be better conceptualised as ‘intercultural’, operating across diverse and shifting cultural frames of reference. Theories identified by this review as useful to guide partnerships include power relations, reflexivity and dialogue, borders and strangeness and the intercultural or third space. This paper examines how these theoretical approaches can develop understanding and improve intercultural engagement between mainstream and Australian Indigenous partners in healthcare.

**Conclusions:**

Rather than viewing partnerships merely as arrangements between disembodied entities, sometimes contractual in nature, they are better seen as activities between people and organisations and essentially dependent on relationships, occurring in an intercultural space that is complex, dynamic and subject to changes in power relations. Theoretical models aiming to understand and improve partnerships indicate the complexity of building and maintaining such partnerships and stress the importance of understanding factors that can strengthen or derail their effectiveness. While the theories presented here are by no means exhaustive, they nonetheless provide a series of entry points through which to engage with the issue and expand the discourse. This approach allows the transformative nature of Australian Indigenous-mainstream ‘culture’ to be explored and understood in its lived expression; rather than relegated to prescriptive categories.

*Partnership is a process that must be recognised as a fundamental part of any strategy for improving health outcomes for Aboriginal people* [1]:48).

The substantial gap in life expectancy between Aboriginal and Torres Strait Islander (hereafter Australian Indigenous) and other Australians has been variously reported as between 11 and 18 years [2]. The health of Australian Indigenous people has been slow to improve despite committed funding [3] and Indigenous Australians remain the most marginalised of any identifiable group, featuring consistently at the lowest point on any marker of disadvantage [4]. As a result of the failure of mainstream services with their Western and biomedical focus to meet Indigenous peoples’ needs, Aboriginal Medical Services were set up in the early 1970s to offer a more culturally safe environment. These provide primary healthcare services run by and for Indigenous Australians to improve access and health outcomes [5,6]. Contemporary Aboriginal Community Controlled Health Services (ACCHS) are a reflection of the aspirations Australian Indigenous people have for self-determination and community control.

While ACCHS remain an important part of the Australian Indigenous healthcare landscape, offering services through separate organisations has not addressed disproportionately high morbidity rates from chronic disease and the social factors impacting on health outcomes [7,8]. The ongoing negative effects of colonisation continue to be reflected in discriminatory practices across sectors including mainstream health services [9-13]. Mainstream health services are defined here as those reflecting the dominant science-based biomedical model of healthcare which is practiced in the developed Western world. These services focus mainly on individual rather than social determinants of health and are based in the view that the scientific model is objective or impartial [14-16]. Evidence of ongoing discrimination or racism against Australian Indigenous people in mainstream health services increases their reluctance to access services and undermines their health outcomes [17].

The terms ‘Australian Indigenous’ and ‘mainstream’ are socially constructed categories with often simplistic notions of two ‘cultures’ or ‘worlds’ [18,19]. The notion that Australian Indigenous people might simply make a choice between two worlds, or simply move between them, selecting the best both have to offer, fails to comprehend the processes through which representations, cultural identities and life-worlds are produced and reproduced [20]. In order to improve Australian Indigenous health outcomes, responsibility must be shared within and across sectors.

Acknowledging the complex social factors contributing to poor Australian Indigenous health has resulted in considerable impetus to form intersectoral and intercultural partnerships to address these issues [21-25]. For example, to address the health impacts of poverty, poor housing and other social and cultural factors, ACCHS link clients to many organisations outside the health sector, often supporting external organisations to deliver services through the ACCHS. However, building such partnerships between Australian Indigenous and mainstream health services can be challenging and complex and are particularly affected by Australia’s historical and current context of race and political relations [25]. Indigenous health in Australia is a highly politicised and contested arena [26], with poor Indigenous health outcomes/indices widely attributed to colonisation and its ongoing expressions [27,28]. With this in mind, mainstream health services must show commitment to fostering ‘genuine, trusting relationships that are tangibly linked to the Aboriginal community’ and avoid a ‘business as usual’ approach if they are to be successful ([25]: 297).

This paper builds on the review conducted by Taylor and Thompson [25] that provides evidence of the importance of partnerships between Australian Indigenous and mainstream health services to improve Australian Indigenous health outcomes. The paper will add to their work by drawing on social theory to offer a framework to inform the establishment and development of such partnerships.

## Background

The term ‘partnership’ has become a catchword to describe almost any type of collaborative relationship between different organisations [29]. Largely borrowed from the business sector [30], it has been embraced as integral to health promotion where ‘multi-sectoral cooperation is the only way of effectively ensuring the prerequisites for health’ ([31]:5, from Jones, [32]). ‘Multi-organisational and community-based partnerships have become dominant social inclusion methodologies, particularly in promoting more joined-up strategies to address cross-cutting community issues’ [33]. As such they reflect a confusing mix of market and collaborative principles.

Variation in the terminology and meaning of partnerships between organisations reflects differences in structure, functioning and goals. Structures may be top down (centrally driven), bottom up (skill development at the operational level), sideways (partnerships between organisations) or community based (grassroots); the type of structure informs how the partnership functions [34]. These variations reflect differences in how organisations share power and control and respond to obligations regarding sharing and disseminating information, [29,35] and this impacts on how partnerships are understood and developed [34]. Partnerships may involve relationships at a variety of levels, between individuals in organisations, organisational management, and individuals and organisations, such that even an Australian Indigenous worker in a mainstream organisation can be said to be in partnership.

A key factor in establishing effective partnerships is clarity of purpose [25,36]. In health service delivery partnerships, ACCHS may variously support mainstream service providers to deliver services or conduct research, use ACCHS’ infrastructure, assist in the delivery of a clinical service and encourage their staff and clients to participate in programs and research studies. Such partnerships also enable planning, training and other development activities to be more cost efficient by pooling or leveraging resources and minimising duplication [37]. Partnerships can be undermined through lack of clarity about respective roles in the partnership resulting in reduced confidence and confusion about the partnership’s purpose, its objectives and how to measure its success. These factors can be compounded at an organisational level by workforce turnover and lack of training and support for Australian Indigenous staff in mainstream health service delivery [25,38]. While the focus of this paper is on intercultural partnerships between Australian Indigenous and mainstream health services, partnering with Australian Indigenous communities is an important part of the process.

### Challenges facing Australian Indigenous and mainstream health service partnerships

Building effective Australian Indigenous and mainstream health partnerships is not without challenges. Politicised and contested [26] national and local tensions both underpin and challenge the fabric of many specific Australian Indigenous-mainstream health service arrangements. Issues commonly reported include: the legacy of Australia’s race relations; ongoing social tensions; the paternalistic role of government and others; disparate ways of working; lateral violence within and across organisations; power dynamics; and funding timeline pressures to deliver outputs that often overwhelm the need to develop and maintain trust and relationships [25,29,39-41].

Partnerships are also affected by issues of governance and representation facing Australian Indigenous organisations. Meshing socio-culturally determined community obligations with service delivery structures whilst satisfying financial and reporting accountability can create complications for Indigenous organisations [42]. The challenge associated with addressing complex problems holistically with limited funds is considerable [29,43]. Community politics also affects Australian Indigenous organisations, a challenge found in many disadvantaged community health programs where the link between those needing or benefiting from an initiative and powerful community ‘gatekeepers’ can be dysfunctional [44]. Finally, little acknowledgement is given to the time needed in the intercultural relational process to establish and build mutual trust that can so easily be undermined by various factors including competing priorities, organisational demands, deadlines and limited resources [25]. Many Australian Indigenous people are disillusioned with what they perceive as empty rhetoric with little action and inadequate funding to support and sustain partnerships [45].

Attempts to improve Australian Indigenous and mainstream relations have often identified points of cultural difference as ‘problematic’ gaps to ‘bridge’ between what were seen as two separate and distinct cultures. The social construction of mainstream Australia is reflected in ‘White Anglo-Australian cultural and racial dominance’ that is considered the ‘invisible omnipresent norm’ – against which Australian Indigenous culture is ‘othered’ and judged ([46]:xix). On the other hand, some of this ‘othering’ comes from Australian Indigenous people wanting their own distinctive history, culture and needs met (see Dudgeon & Fielder [27]). However, using simplistic concepts of culture runs the risk of essentialising cultures into fixed or immutable characteristics that are represented in opposition to each other [47,48]. Another pitfall is constructing Australian Indigenous culture as mono-cultural thereby negating the diversity between cultural groups within communities as well as across the country [18-20,49]. Attempting to understand Australian Indigenous-mainstream health service interactions through essentialised cultural representations that reinforce difference, denies the complexity of the intercultural terrain and can reproduce stereotyping of cultural groups [50]. Not surprisingly, this can erode the effectiveness of intercultural partnerships.

### Strategies to improve Australian Indigenous-mainstream health service partnerships

The complex and tenuous socio-political arena of Australian Indigenous and mainstream health partnerships invites theoretical approaches to help understand the terrain and inform practice. Literature on theoretical approaches informing successful intercultural partnerships in the health sector is limited [51,52]. While there are prescriptive recommendations about successful ways to develop Australian Indigenous and mainstream partnerships, organisations applying these run the risk of negatively impacting on the effectiveness of partnerships if limited attention is given to the complexity of the terrain [53]. Identifying theoretical models that inform effective Australian Indigenous and mainstream partnerships in healthcare can limit the prevalence of ad hoc approaches, challenge reductionist attitudes that distances individual health from the wider social context, and respond to questions about why theory is needed at all [54,55]. Without theory to guide us, our capacity to understand factors informing effective Australian Indigenous and mainstream partnerships remains limited [34]. Healthcare organisations that use theory to inform practice can develop their understanding of this complex terrain with a view to improving Australian Indigenous healthcare and health outcomes.

## Method

Rather than viewing partnerships merely as arrangements between disembodied entities, sometimes contractual in nature, this review was premised on two broad philosophical perspectives. The first is that partnerships are arrangements between people. Even when the partnerships are between organisations, potentially articulated through contracts and agreements, partnerships are essentially dependent on relationships between people. Thus, theoretical models aiming to understand and improve partnerships through a key focus on managing the inter-relational domain are fundamental. The second is that the intercultural space is complex, dynamic and subject to sudden changes in power relations.

The literature search is a crucial initial step requiring a degree of familiarity particularly where the literature to be reviewed is concerned with complex, under-researched issues, as keywords may not be clearly indexed and primary sources may be less accessible. Therefore it is important to work with ‘information experts to develop sensitive and specific search strategies’ [56]. Greenhalgh et al. [57] suggest using informal and unstructured methods such as networking with experienced practitioners and social researchers from the field as an initial ‘territory mapping’ exercise [57] in order to gain a feel of the overall literature before developing search terms when investigating an underdeveloped area. Once this early mapping is in place, references that were seen as influential by researchers can be identified.

For this review, an initial workshop was convened with a group of six researchers and health professionals from a variety of disciplinary backgrounds, all with experience and academic expertise in Indigenous Australian health. Participants considered and discussed relevant terms and frameworks that related to Indigenous Australians and mainstream health partnerships and to working in the intercultural space; these terms were then discussed and added to by other experts and the resultant list was used as the starting point for a broad data base search for relevant articles. Several electronic data bases were searched to source literature on the topic including: Scopus, Web of Knowledge, Cochrane Library, AIHW's Closing the Gap Clearinghouse, Centre for Aboriginal Economic Policy Research (CAEPR), Health and Medical Complete, Health and Society, JAMA & Archives, Meditext, MEDLINE, PILOTS, PsychINFO and PubMed.

Key terms to identify relevant articles included but were not restricted to: partnership; Aboriginal cross-cultural partnerships, Aboriginal organisation mainstream partnerships, Indigenous Australian organisations partnerships, Aboriginal mainstream engagement, partnership theory, intercultural partnership, Indigenous partnership, Aboriginal partnership, Aboriginal and Torres Strait Islander partnership, mainstream partnership, health service[s] partnership, intercultural relationship, intercultural space, border theories, power relations, reflexivity, strangeness and dialogic theory. As emerging theories focused the enquiry, the search became progressively more systematic and purposive sampling strategies such as ‘snowballing’ search techniques (searching references of references and using citation tracking software) were used to identify later papers that had cited a seminal source.

A substantial literature on partnerships was identified (669 papers) and considered for its relevance to Indigenous Australian health contexts, mainstream Australian health contexts, intercultural contexts and selected social theories. As suggested by Whitemore [58], an integrative approach, bringing together theoretical methods with practical considerations about working in partnership, informed the analysis of the literature. Integrative reviews and a condensation of the literature [59] enable knowledge themes to be revealed to offer a more comprehensive and refined understanding [58] of an issue. Further synthesis of the literature then enabled key theoretical concepts to emerge [60].

## Findings

The synthesis of the reviewed literature identified six key theories or theoretical concepts, all integral to partnerships that operate in Australian Indigenous and mainstream healthcare contexts. In the following sections we look first at power as a key theoretical starting point. This is followed by an exploration of how reflexivity and dialogical theory can guide the process of understanding partnerships by linking theory to practice. Two further useful theoretical concepts, ‘strangeness’ and ‘borders’, are then investigated before a final section elucidating a theory of the intercultural space.

### Power relations

Complex and often ambiguous, power is strongly emphasized as an instrumental point of analysis in the intercultural exchange [61,62]. Power inequalities are central to the continuing effects of race relations in countries colonised by Western nations [42]. In any social exchange, power sets the limits and affords the possibilities for action [63].

Given the legacy of colonisation, Australian Indigenous actors may be hyper alert and suspicious of (colonial) mainstream control, which can become evident when participating in a partnership [42]. Therefore, at the heart of an intercultural partnership attempting to negotiate power, is trust. Inter-organisational and interpersonal trust remains integral to partnership performance [64]. However, while trust is at the heart of a succcessful, intercultural partnership in Australian Indigenous health, it can be compromised by many factors, not least inequitable power relations [17,25,65]. While partnerships between mainstream and Australian Indigenous health services may often be ‘acutely conscious of the power relations that exist … and try hard to reduce the power imbalance that seems inevitable’ ([29]:51) , this can be easily undermined by systemic factors including the cessation of funding to projects involving Australian Indigneous and mainstream health service partners ([29]:51).

Examples abound where partnerships have been unsuccessful in building trust or achieving equity [62]. Tesoriero [30] suggests that aspiring to trust and equality, yet failing to recognise the reality of lived experience and plan accordingly, can lead to failure; pursuing ‘maximum trust levels in a partnership maybe self-defeating, setting up expectations that cannot be achieved’ ([30]:52). Applying a more nuanced approach to power, Tesoriero describes a partnership characterised by mistrust and inequality that was nonetheless sustained over a long period [30]. From his analysis of power in this partnership, Tesoriero found that aiming for equal power may not be the key to successful partnerships. Instead, how partners use the power they have is more important, recognising that as well as ‘power over’ there is also the ‘power to achieve’. For example, perseverance may represent a sophisticated use of what little power a partner has. That is, the ‘sense of powerlessness’ identified by a less powerful community group was also used successfully to invoke a social justice response from the more powerful partner [30].

Purdy’s [52] framework for analysing power in partnerships uses a matrix of sources of power (authority, resources and legitimacy) and arenas or opportunities to exercise power (participants, process and content). This framework describes ‘the kinds of power held and how they can be exercised structurally and relationally in collaborative processes’ ([52]:410). It recognises both overt and more subtle use of power and helps overcome self-serving biases in assessing power, thus can ‘reveal mistaken beliefs and hidden sources of power that may reduce over-confidence, defensive or domineering behaviours during collaborative processes’([52]:415).

Bourdieu’s theory of social practice offers a framework to understand power relations and the multiple, fluid and dynamic forces influencing intercultural partnerships [66-68]. He argues that while objective structures such as class (or culture) influence how individuals relate, they are also influenced by the structures or organising principles of a particular ‘field’ – in this case, the healthcare field ([66]:73; [69]:17). Bourdieu ([69]:17–19) describes a socially organised space—such as healthcare—as a ‘field’ of conflict and competition. Individuals in this field, depending on the position they hold, define who has the power and struggle either to keep or change the boundary of that field. How intercultural partners negotiate this tension is a key element in whether the boundaries of the healthcare field change.

Cultural differences around governance structures and decision making can result in inter as well as intra-cultural tensions ([19]:1). These tensions reflect the ‘deep structural legacy of the colonial encounter' [70] that impacts not just on partnerships but also on service delivery. Bourdieu ([69]:19) argues that individuals or ‘agents’ must think reflexively if they want to challenge the boundaries of a particular field. Yet the complexities of the structure and function of intercultural partnerships [67] often determine, permit or constrain the agency of individuals. Individuals within an intercultural partnership act not only from their internalised cultural and social domains but also in response to how their cultural identities are externally represented [20].

### Reflexivity and dialogical theory

Processes around negotiating power and building trust need to be approached with caution. Not recognising the reality of lived experience and strategising accordingly, can lead to failure as suggested by Tesoriero [30]. Instead, reflecting on intercultural practice, the complex tensions involved, and devising appropriate strategies to respond, can foster creativity and offer a more positive and strengths-based approach to the dynamic potential of developing Australian Indigenous and mainstream health service partnerships.

The process of reflexivity involves self-reflection as a first step. Reflecting on various factors including history, social influences, family experiences and individual beliefs and values that inform how we interact in intercultural relations can reveal what assumptions we project on to the ‘other’ [71]. That is, to better understand the other, the focus must be first on understanding and ‘problematizing’ ourselves [72], exploring our own positioning in intercultural relations and how that informs our understanding of the ‘other’.

In the Australian context and referring in particular to non-Indigenous health practitioners working within an Indigenous setting, the emotional response of individuals to guilt needs to be explored. Naming and understanding the reasons for guilt can provide individuals with an opportunity to work through feelings of shame and distress over Australia’s historical and current relationship with Indigenous people [73]. For example, Kowal and Paradies argue that many ’Whites’ working among Australian Indigenous people tend to be liberal-thinking, left-leaning, ‘anti-racist’ [26]. Yet, Kowal’s later research on progressive White, anti-racist health professionals working in Indigenous Australian health [74] found they were self-effacing and reluctant to claim any agency for improving Australian Indigenous health. The complexities of this ‘political correctness’ can create practitioner ambivalence about their ‘helper’ identities that can lead to inaction [26]. Kowal et al. [75] suggest that White health professionals who develop a reflexive, anti-racism view also avoid essentialising cultures into ‘good’ and ‘bad’, acknowledge their Whiteness and recognise the benefits they gain without being immobilised by guilt and anxiety. This can be facilitated by effective anti-racism programs that offer tools to manage guilt and anxiety and avoid inaction [75]. In this way, awareness of power differentials does not necessarily lead to inactivity [26], and personal transformation and social change—which are closely connected—may occur.

Reflexivity can also be applied by organisations, as there are differences between trust in individuals and trust in institutional patterns of belief, values and behaviour [76]. For organisations in formal partnerships, reflexivity may involve understanding how group identity has been constructed in response to an externally located cause [67] and the implications of this for partnerships. Australia’s history includes injustice to Indigenous Australians and this means collective defences are complex and discussions of guilt are difficult to separate from interactions [73].

Reflexivity for many Indigenous Australians has recently involved examining the deficit mindset within Indigenous Australian communities that produces a lateral violence that, among many things, targets Indigenous identity, and affects developments in health, society and economy. Lateral violence may not necessarily express itself as overtly harmful behaviours, but rather an underlying fear and anger [77] towards other community members that constrains relationships [78].

Dialogical theory supports the reflexive process as a tool to explore how each interaction builds on the one before; thereby shaping and reshaping the intercultural encounter [79,80]. While partnerships may have formalised endeavours, intercultural partnership relations are shaped by the interactive nature of relationships [79,80]. That is, interactions continually respond and expand on previous interactions or enter into dialogue, suggesting that building understanding and engaging with difference is an iterative process [81].

Given that intercultural exchanges occur in social contexts, multiple dialogues and multiple voices can happen simultaneously and influence both individual and organisational exchanges [82]. Individuals within organisations who use a dialogic approach to explore partnership commit to genuine and ongoing questioning of their positioning – “What position do I hold individually, and what do I represent at an organisational level?”; “How do these positions change when our partnership engages?”; “How does it influence the partnership in action?” [63,71]. Thus, using dialogics offers partners the opportunity to explore potential points of resistance experienced in their partners and themselves (as the ‘points of difference’) [71].

### Strangeness and borders

The reflexive process enabled by dialogical theory can support addressing power relations and building trust. Two theoretical concepts are useful here – ‘strangeness’ and ‘borders’. Strangeness refers to both the ‘experience of strangeness’ and the ‘position of stranger’ [71], where individuals or groups experience degrees of ‘strangeness’ when physically close to someone, but socially and culturally distant [71]. The ambivalent position of the stranger can cause confusion and anxiety in the dominant culture, as she/he makes explicit the porous nature of social boundaries [71]. Experiencing difference as strangeness may be emotionally charged and this is often behind intercultural conflict [83]. However, understanding can be built within the intermediate space between familiarity and strangeness [83] as recognising alternative (strange) knowledge is a critical pedagogical decolonising process [84].

Similarly, the ‘stranger’ can question the “taken for granted” world of the host and this uncertainty creates an opportunity for a critical understanding of the host’s world. In this sense, Indigenous Australian actors and/or organisations partnering with mainstream bodies, by their position as ‘strangers’ in a white-dominated society, offer potential to critically examine partnerships by challenging dominant systems and paradigms [84]. Recognising strangeness is also important in the social and political environment as the experience of strangeness impacts on policy decisions.

Distinctions and differences are essential to the human condition. Constructing borders is an innate human process helping us to organise our world; “…it is how we deal with these differences that make boundaries menacing and oppressive or liberating and empowering” [71]. Border work therefore must simultaneously explore the necessary construction of certain boundaries, as well as their permeability, without those boundaries being stereotyped as cultural ‘attributes’ while working to construct fluid boundaries across cultures rather than solid borders that divide us [53,85]. It is through engagement with ‘difference’ and the construction of boundaries that individuals in the intercultural domain have the opportunity to reflect on their own preconceptions [83].

Border theories may offer partnerships a platform for unpacking how, and why, individuals and organisations construct and maintain certain inter-relational boundaries, evident particularly in negotiating shared partnership activities [53]. This might involve inviting partners to collaboratively explore what boundaries need to be maintained and why, where there is the potential for ‘crossing’ and what aspects within the partnership could potentially facilitate or hinder a crossing or successful intercultural exchange. That is, partners attempting to ideologically deconstruct their boundaries may not only be unrealistic but may overlook the usefulness and necessity of group and individual identity construction [53]. Understanding borders is an instrumental process for work in the collaborative intercultural zone and ‘mapping’ these frontiers is an important part of understanding what is going on [53]. Intercultural relations are shaped as much by what the margins define as the liminal points of translation and transformation.

Theoretical concepts of power, strangeness and borders, examined through the reflexive lens demonstrate that Australian Indigenous and mainstream relationships, understandings and practices are defined by various micro and macro forces that can be transformed within the intercultural field [82] rather than an arrangement occurring between separate, definable cultural domains [20,49]. With this in mind, elucidating a theory of this intercultural space is key [72]. The diversity and fluidity of the intercultural space reflects its relevance and complexity when conceptualising and developing intercultural partnerships [86].

### The intercultural space

Homi Bhabha famously coined the term ‘third space’ to describe the liminal, intercultural space between cultures [48]. Bhaba ([48]: 208) cautioned that in societies where cultural diversity is encouraged, discrimination or racism is ‘rampant’. He argued that the universalism that paradoxically permitted diversity ‘masks ethnocentric values, norms and interests’. Bhabha rejected the idea of a purely oppositional space where one culture dominated another. Instead he suggested everything happens in between. This ‘third space’ requires different ways of relating so other positions, views and identities can emerge through interaction, negotiation and translation between, for example, the ‘colonizer’ and the ‘colonized’ [87]. Through collaboration, each has the opportunity to disrupt colonial narratives and create new possibilities [87]. Nakata [88] suggests that each can engage in rigorous discussion to share ideas, experiences and understandings at the cultural interface. This can result in a struggle between two knowledge systems ‘where things are not clearly black or white, Indigenous or Western’ ([88]:9). While holding tension in this space can be risky, it can also result in the emergence of new ideas and positions [89].

Instead of positioning cultures in opposition to each other, the intercultural space is understood as a layered and complex entanglement of concepts, theories and sets of meanings ([88]: 272). Bhaba [48] and Nakata et al’s [88,89] concept of the intercultural space avoids essentialism and moves cultural differences from being a ‘problem’ to a more powerful discourse that promotes sharing of ideas and robust discussion that engages rather than resists cultural differences [62]. Langton suggests that the intercultural space has the potential to encourage individuals or agents to test and repeatedly readjust preconceptions of the ‘other’ as a strategy to better understand each other [90]. This includes partners critically reflecting on factors that inform their own cultural and racial positioning and its impact on sustaining or undermining the partnership. While this may be challenging, Emirbayer and Desmond ([91]: 514) suggest that:

...our understanding of the racial order will remain forever unsatisfactory so long as we fail to turn our analytic gaze back upon ourselves, the analysts of racial domination, and inquire critically into the hidden presuppositions that shape our thought.

These six theoretical approaches for working in the intercultural space described above are summarised in Table 1. In addition to brief descriptions, each approach is also considered in terms of how it is applicable at the individual and the organisational level.

**Table 1:** **Theoretical approaches for working in the intercultural space**

1. **Power relations**	
• In any social exchange, power sets the limits and affords the possibilities for action [63].	
• Power manifests in communication and language	
• The struggle for power in the intercultural domain is not static: it is always shifting, generally asymmetrical and may be obvious or subtle [63].	
• Power is strongly emphasised as an instrumental point of analysis in the intercultural exchange [61].	
• Power is often driven by the movement of resources, particularly funds.	
• Although asymmetrical power in the intercultural domain can be a centrifugal force, it can co-exist with the potential for centripetal action founded on commonalities of the human condition [67].	
• Unequal power distribution is an ongoing legacy of colonialism that still deeply influences Indigenous-non Indigenous relations. Accordingly, indigeneity continues to be “defined and self-defining in terms given by the more powerful, colonising and, in many different ways, often racist white population” [61].	
• The politics of cultural identities in governance processes is best understood through an intercultural lens, in terms of power and political identity [86].	
• Indigenous governance must be understood as having a crucial role as a counter to mainstream governance.	
**Individual (agency)**	**Organisation (structure)**
• Indigenous actors may be hyper-alert and suspicious of (colonial) mainstream control, which can become evident when they participate in a partnership [42].	• Power relations differ depending on the type of partnership.
	• Inter-organisational and interpersonal trust is at the heart of power negotiations in an intercultural partnership. Trust is a key basis for partnership performance [64].
• It is important to distinguish between trust in individuals and trust in institutional values and mores [76].	• Power is a crucial issue confronting Indigenous groups due to the intercultural space in which Indigenous organisations are inherently operating [82].
	• It is important to consider the most effective strategies for building trust in institutions, and which are supported by government [92].
2. **Reflexivity**	
• Reflexivity involves exploration of self-positioning, to understand the lenses that influence our understanding of one another.	
• Operating in the intercultural space is affected by history, social influences, family experiences and personality, and thus the reflexive encounter with one’s self can reveal qualities projected on to the ‘other’ [71].	
• Kowal and Paradies argue that many ‘whites’ working among Indigenous people tend to be liberal-thinking, left-leaning and ‘anti-racist’ [26]. The complexities of this ‘political correctness’ can overemphasise structure and downplay agency, creating ambivalence and humility in practitioners about their ‘helper’ identities, and ultimately result in inaction [26].	
• Reflexivity for many Indigenous Australians has recently involved examining the deficit mindset within Indigenous communities that produces a lateral violence that, among many things, targets Indigenous identity, affecting developments in health, society and economy.	
• Australia’s history of injustice means that collective defences are complex, and that discussions of guilt are difficult to separate from interactions [73].	
• Lateral violence may not necessarily express itself as an overt racism but rather an underlying prejudice that constrains relationships—whether between ‘white’ and ‘Indigenous’ governance structures, or service providers and clients, or just between individuals [78].	
**Individual (agency)**	**Organisation (structure)**
• To better understand the other, the focus must ultimately be first on understanding, and ‘problematising’ ourselves [72]. In this sense, social change and personal transformation are closely connected.	• For organisations in formal partnerships, reflexivity may involve understanding how group identity has been constructed in response to an externally located cause [67]
• The emotional response of individuals to guilt needs to be explored further. Naming and understanding the reasons for guilt can result in and create a transformational opportunity for change by enabling individuals to begin working through it [73].	
• Kowal and Paradies argue for a ‘reflexive anti-racism’, where awareness of power differentials doesn’t lead to inactivity [26].	
3. **Dialogical theory**	
• Dialogical theory suggests that each interaction builds on the one before, thereby shaping and reshaping the intercultural encounter [79,80]	
• Interactions constitute continual response to and expansion on previous interactions, that is, interactions form a dialogue	
• Dialogical theory suggests understanding and engaging with difference [81].	
• Intercultural exchanges are (or may be) socially orientated rather than structural, and as such, multiple dialogues with multiple voices can happen simultaneously. It is important to understand the impact of social influences on individual and organisational exchanges [82].	
**Individual (agency)**	**Organisation (structure)**
• The self is understood as ‘culture-inclusive’ and culture as ‘selfinclusive’, with a dynamic relationship occurring that does not see self (the individual) as separate from culture: rather they are a composite of parts [81].	• While intercultural partnerships may have formalised endeavours, they are shaped by the interactive nature of relationships [79,80].
• When individuals within organisations explore a partnership through a dialogical approach, there must be commitment to genuine and ongoing questioning of positioning: “What position do I hold individually, and what do I represent at an organisational level?”; “How do these positions change when our partnership engages?”; “How does it influence the partnership in action?” [63,71].	• Using dialogics offers organisations the possiblity of exploring potential points of resistance (the ‘points of difference’) [71].
4. **Borders**	
• Constructing boundaries is an innate human process, helping us to organise our world [71].	
• Border work must simultaneously explore the necessary construction of certain boundaries, as well as their permeability, without those boundaries being stereotyped as cultural ‘attributes’. That is, border work means constructing fluid boundaries across cultures, rather than solid borders that divide us [53,85].	
• Distinctions and differences are essential to the human condition; “it is how we deal with these differences that make[s] boundaries menacing and oppressive or liberating and empowering” [71].	
• Intercultural relations are shaped as much by what the margins define as by the liminal points of translation and transformation.	
• Understanding borders is an instrumental process for work in the collaborative intercultural zone, and ‘mapping’ these frontiers is an important part of understanding what is going on [53].	
**Individual (agency)**	**Organisation (structure)**
• It is through engagement with ‘difference’, and the construction of boundaries, that individuals in the intercultural domain have the opportunity to reflect on their own preconceptions [83].	• Border theories may offer partnerships a platform for unpacking how, and why, individuals and organisations construct and maintain certain inter-relational boundaries, evident particularly in negotiating shared partnership activities [53].
• Through reflection, prejudices can potentially be productive in creating understanding [83].	
• Partners attempting to ideologically deconstruct their boundaries may not only be unrealistic but may overlook the usefulness and necessity of group and individual identity construction [53].	• Consideration of these theories prompts partners to collaboratively explore what boundaries need to be maintained and why, where there may be potential for ‘crossing’, and which aspects within the partnership could potentially facilitate or hinder a crossing or successful intercultural exchange.
5. **Strangeness**	
• Refers to both the ‘experience of strangeness’, and the ‘position of stranger’ (an individual or group different to the majority) [71].	
• The ambivalent position of the stranger can cause confusion and anxiety in the majority world, as they make the porous nature of social boundaries explicit [71].	
• Understanding is built within the intermediate space between familiarity and strangeness [83].	
• Recognising that there are alternative (strange) knowledges is also a critical pedagogical decolonising process [84].	
• The stranger can question the ‘taken for granted’ world of the host and this uncertainty creates an opportunity for a critical understanding of the host’s world. In this sense, Indigenous actors partnering with mainstream bodies, by their position as minority and strangers in a white-dominated society, offer potential to critically examine partnerships.	
**Individual (agency)**	**Organisation (structure)**
• Individuals experience degrees of ‘strangeness’ when physically close to—but socially and culturally distant from—someone [71].	• Dominant systems and paradigms should be interrogated [84].
• The experience of differences as ‘strangeness’ may be emotionally charged, and this often underlies intercultural conflict [83].	• Recognising ‘strangeness’ is also important in the social and political environment, as the experience of ‘strangeness’ impacts on policy decisions.
**6. The Intercultural Space**	
• Recognises the significance of the relational and situational context of communication [76,101]	
• Recognises that apprehension and ethnocentrism can negatively affect the ability to communicate effectively and to reduce uncertainty [102]	
• Examines the inter-relationships between uncertainty, anxiety, mindfulness, and communication effectiveness [103]	
• Describes the complexity of key positions of activity in the intercultural field (from the ‘silent majority’ and ‘spectators’ through to leaders and external stakeholder—each with their own ‘domain’ of cultural activity), not only interculturally but cross-sectorally and interprofessionally [104].	
• Individual psychological and emotional responses to relations in an intercultural exchange can be a centrifugal force or can serve productively to establish necessary boundaries.	
**Individual (agency)**	**Organisation (structure)**
• Individuals are encouraged to shift focus from reducing negative emotional responses (such as anxiety) to managing them, according to individual capacity [103].	• Organisations can support individuals to identify skills, behaviours, competencies and attributes that can assist them in their intercultural interactions.
• Capacity is defined by cross-cultural and intercultural competencies including:	• Organisations should encourage reflection on organisational culture as the context for communication.
- the ability to interpret and relate;	• Building intercultural relationship skills among key stakeholders is critical to working more effectively (as highlighted by a case study of a successful intercultural partnership in Queensland [104]).
- the ability to have a positive and curious approach to differences and unfamiliarity;	
- the ability to be sensitive to human diversity and to have insights about how culture influences communication and language;	
- open to ‘otherness’;	
- a fluid and hybrid identity and anti-essentialist notions that strongly incorporate the concepts of mindfulness [63,71,105]	

### Intercultural partnerships in practice - applying theory

While the experience of tension in intercultural partnerships can bring partners together in discussion and sharing of ideas that can lead to new understandings, it can also drive them apart. One example involved an Australian Indigenous-mainstream partnership with a joint management arrangement in Kakadu National Park in Australia’s Northern Territory where contradictory forces were described:

… centripetally, drawing Indigenous and white actors together and reinforcing cohesion; and the centrifugal forces that define the two groups, define and maintain their boundaries, and threaten to dissolve the nexus of joint management potentially creating antagonistic chasms [67]:180).

Australian Indigenous and mainstream partnerships can break down because of centrifugal forces driven by top-down approaches requiring partnerships under statute to become incorporated. This can lead to conflict when previously partnerships have been more fluid [49]. Centrifugal and centripetal forces can arise concomitantly from the same source such as government policies and funding; these may facilitate centripetal forces that are reflected in partners with similar visions at the beginning of the partnership which, once established, change as different values and beliefs surface resulting in a centrifugal effect [98].

This process links to Bhabha’s notion of the intercultural space being one of struggle but also with potential for change [48]. The tension in the struggle creates opportunities for interaction that may result in new behaviours and ways of being [98] that can be both positive and negative; both centripetal and centrifugal. The challenge, according to Nakata et al. [89], is to avoid the familiarity of resorting to binary cultural oppositions that shut down inquiry and limit understanding but instead engage in discussion and negotiation to explore new ideas and ways of working. This might include Indigenous Australians telling mainstream health providers about their knowledge and culture instead of conceptualising it through a ‘Western scientific filter where it is disembodied from its people’ ([88]:9). While this might be uncomfortable and create resistance and tension for mainstream health providers, adopting an approach that acknowledges and values Australian Indigenous culture, history and lived experience opens the door to a:

…discourse of possibility, where the missing voices and knowledges can be heard and validated … [in] a … system that is both more inclusive and better able to respond to the varied multiple knowledges’ ([99] :194).

In what has been termed ‘the discomfort zone’ of intercultural work, the productive potential of difference is emphasised and ‘the necessary work of choosing to put oneself in that space’ and as such, ‘in the discomfort zone of cultural contact, the actual work of collaboration was achieved’ ([53]:264–265). This suggests that an intercultural partnership will often experience some discomfort where beliefs are challenged, yet it provides an opportunity for health providers and the services in which they work to reflect on factors that undermine or strengthen such partnerships. If mainstream health services are committed to working in the intercultural space, they must expect to ‘initiate organisational change to improve their capacity to work in partnership with a wide variety of communities’ ([100]:855).

A key factor in establishing partnerships is clarity of purpose. If the purpose of building partnerships between the Australian Indigenous and mainstream health services is to improve Aboriginal and Torres Strait Islander health outcomes, collaboratively identifying ways to do this is important. This might include two-way, cross-cultural learning to facilitate the more effective delivery of comprehensive and culturally congruent services [1,22,23,39,40,101-105]. Mainstream health services might offer professional development, such as education and training in culturally safe healthcare, that concomitantly leads to increased access to services by Australian Indigenous people while acknowledging and building staff capacity to respond to the broader social determinants of Australian Indigenous health [25].

One effective strategy to improve Australian Indigenous and mainstream health service partnerships is to establish a partnership based on the needs articulated by the Australian Indigenous community that allows enough time and resources to collaborate and develop trust [25]. This might include meeting regularly and getting to know each other, developing skills in problem solving to effectively manage cultural differences and/or conflict between partners. This approach also requires organisational commitment to adopt an equitable approach to power sharing that, at a practical level, might include alternating the chairing of meetings and location of meetings [25].

## Conclusion

This paper draws on social theory to inform the establishment and development of intercultural partnerships between Australian Indigenous and mainstream health organisations. While the theories presented are by no means exhaustive, they nonetheless provide a series of entry points through which to engage with the issue and expand the discourse. In particular reflexivity and dialogical theory are essential theoretically informed ways to work in practice that ensure attention is paid to the nature of partnerships in terms of power, strangeness, borders and intercultural relations.

While this review focuses on health service partnerships, the theories described here may be applicable to other partnerships that aim to improve the health of Indigenous Australians. These theories highlight the complexity of building and maintaining such partnerships and stress the importance of understanding factors that can either strengthen or derail their effectiveness. The theories selected offer a layered, more textured approach to guide our understanding and help address the challenges that participants (and the organisations they work within) face in intercultural partnerships, recognising the benefits that working through any problems that occur. From this place of struggle, where partners withstand the tension rather than resort to the familiarity of binary cultural positioning, new ways of working in intercultural partnerships can emerge. Finally, this review implies the need for further research to test the application of these theories to practice.
